# Diversity protects plant communities against generalist molluscan herbivores

**DOI:** 10.1002/ece3.359

**Published:** 2012-08-31

**Authors:** Yvonne Fabian, Nadine Sandau, Odile T Bruggisser, Patrik Kehrli, Alexandre Aebi, Rudolf P Rohr, Russell E Naisbit, Louis-Félix Bersier

**Affiliations:** 1 Unit of Ecology and Evolution, University of FribourgChemin du Musée 10, CH-1700, Fribourg, Switzerland; 2 Station de recherche Agroscope Changins-Wädenswil ACWCH-1260, Nyon, Switzerland; 3 Laboratory of Soil Biology, University of NeuchâtelEmile-Argand 11, CH-2000 Neuchâtel, Switzerland; 4 Integrative Ecology Group, Estacion Biologica de Doñana EBD-CSIC41092, Sevilla, Spain

**Keywords:** Agroecosystem, biodiversity, ecosystem functioning, gastropoda, herbivory, plant composition, resource concentration hypothesis

## Abstract

Wildflower strips are used to increase natural enemies of crop pests and to conserve insect diversity on farmland. Mollusks, especially slugs, can affect the vegetation development in these strips considerably. Although recent theoretical work suggests that more diverse plant communities will exhibit greater resistance against herbivore pressure, empirical studies are scarce. We conducted a semi-natural experiment in wildflower strips, manipulating trophic structure (reduction in herbivorous mollusks and reduction in major predators) and plant diversity (2, 6, 12, 20 and 24 sown species). This design allowed us to assess the effect of plant diversity, biomass and composition on mollusks, and vice versa, the effect of mollusc abundance on vegetation. Seven species of mollusks were found in the strips, with the slugs *Arion lusitanicus*, *Deroceras reticulatum* and *Deroceras panormitanum* being most frequent. We found a negative relationship between plant diversity and mollusk abundance, which was due predominantly to a decrease in the agricultural pest species *A. lusitanicus*. These results are consistent with the hypothesis that plant diversity can reduce the impact of herbivores. However, plant identity also had an effect on mollusks, and accounted for a much larger fraction of the variation in mollusk communities than biodiversity effects. While overall plant diversity decreased during the 3 years of the study, in the final year the highest plant diversity was found in the plots where mollusk populations were experimentally reduced. We conclude that selective feeding by generalist herbivores leads to changes in plant community composition and hence reduced plant diversity. Our results highlight the importance of plant biodiversity as protection against generalist herbivores, which if abundant can in the long term negatively impact plant diversity, driving the system along a “low plant diversity – high mollusk abundance” trajectory.

## Introduction

Declining global biodiversity has inspired a large number of studies analysing the effects of plant diversity on the diversity and abundance of higher trophic levels and on ecosystem functioning (e.g.,Tilman et al. 1997; Cardinale et al. 2006; Haddad et al. 2009; Scherber et al. 2010a). Two contrasting hypotheses focusing on plant-herbivore interactions have been formulated. The *more individuals hypothesis* (Srivastava and Lawton 1998) suggests that diverse plant communities are often more productive than simple plant communities (Tilman et al. 2001) and provide a greater quantity of resources for consumers, thereby increasing their number. Furthermore, herbivores may also increase their consumption and biomass when feeding on a more diverse plant community, as is the case in grasshoppers (Pfisterer et al. 2003; Unsicker et al. 2008). Whereas the *more individuals hypothesis* assumes a similar effect on all herbivore species, the *resource concentration hypothesis* (Root 1973) makes a prediction only for specialist herbivores: specialist populations are expected to increase when their food plants are at high abundance. Therefore, species-poor plant communities should show higher specialist herbivore abundances than diverse plant communities where host plants are more dispersed. Hence there is a lower risk of specialist herbivory in species-rich plant communities. The situation is less clear for generalists, although they can also show feeding preferences (Scherber et al. 2010b) and thus should respond to changes in plant composition.

Vegetation characteristics other than plant diversity and composition are also important for the abundance and species richness of herbivores. For example, dense vegetation may serve as hiding-place from enemies (Jeffries and Lawton 1984), cover from the sun (Archard et al. 2004), or nesting place (Briner et al. 2005). High plant biomass may also ensure high food availability and cover over time. The *plant architecture hypothesis* (Lawton 1983) states that the physical structure of the aerial parts of the host plant influences the community structure of herbivorous insects, resulting in greater herbivore abundances in stands with more complex structure and greater biomass (Riihimaki et al. 2006; Randlkofer et al. 2009). However, the relative importance of plant diversity, composition and structure for the herbivore community in natural ecosystems has not been quantified in earlier studies.

The herbivore community can, in turn, affect plant diversity and community composition by selectively feeding upon particular species and altering competitive interactions (Buckland and Grime 2000; Buschmann et al. 2005; Howe et al. 2006; Scherber et al. 2010b; Allan and Crawley 2011). Herbivory can affect plant diversity positively, negatively, or neutrally, depending on the herbivore species and habitat type. Herbivorous mollusks like slugs are known to alter plant species richness and composition, by selectively feeding on plant seedlings. They also have the potential to alter plant biomass, as has been shown in microcosm experiments (Buckland and Grime 2000; Buschmann et al. 2005; Lanta 2007). However, the effect of mollusks on the vegetation of species-rich natural ecosystems is less well understood (but see Hanley et al. 1995; Allan and Crawley 2011).

Due to intensification of agriculture, a drastic loss of biodiversity has occurred in agro-ecosystems in the second half of the 20th century (Kruess and Tscharntke 1994; Tscharntke et al. 2005). To counter species decline, agro-environmental schemes were introduced across Europe, with payments to farmers and other landholders to address environmental problems or to promote environmental amenities (OECD 2003). More than a decade has passed since their introduction, and studies of the ecological effectiveness of such schemes have shown both positive and negative impacts (Kleijn and Sutherland 2003; Knop et al. 2006; Haaland et al. 2011). For farmers, benefits include the establishment of pollinators and biological control agents (Haaland et al. 2011; Pywell et al. 2011), but there is also the risk that they will foster herbivorous pests such as mollusks (Frank 1998a) or voles (Briner et al. 2005).

Wildflower strips are one form of agro-environmental scheme. In the Swiss lowlands they are made up of a recommended wildflower mixture containing 24 herbaceous species (Schaffner et al. 1998) sown inside agricultural fields or along their edges, and maintained for 6 years (Nentwig 1992). The wildflower species were chosen to benefit a maximal number of taxa, including arthropods that play an important role in pollination (Carvell et al. 2007) and biological control (Nentwig 1992). However, the strips are also favorable habitats for mollusks (Briner and Frank 1998; Keller et al. 1999; Günter 2000; Frank 2003) and micromammals (Aschwanden et al. 2007), because several of the plant species included are eaten by these groups and also provide dense cover, which offers reproduction sites and protection (Briner and Frank 1998). Severe slug damage of crops adjacent to wildflower strips has been recorded, especially by *Arion lusitanicus* Mabille and *Deroceras reticulatum* Müller (Frank 1998a). *Arion lusitanicus* is native to Southern Europe but is now invasive across Europe (Schmid 1970). It prefers open areas and has become a severe pest in arable land in the last decade (Frank 1998b; Grimm 2001). In some habitats (wildflower strips and meadows) densities of more than 50 individuals per square metre have been observed (Grimm 2001). Slugs of the genus *Deroceras* are pests in agricultural areas all over the world (Clemente et al. 2008), but are native to central Europe (Kerney et al. 1983).

The mollusk community in wildflower strips and, in particular, its relationship with plant composition, diversity and structure is, to our knowledge, poorly understood. In a 3-year experiment where sown plant number and mollusk abundance were manipulated, we first studied the importance of plant diversity, structure, and composition on the abundance of mollusks and especially slugs; secondly, we estimated the effect of mollusks on the plant community. We addressed the following specific questions: (1) Are species-rich plant communities more resistant to mollusk invasion than species-poor communities? (2) Is vegetation structure, plant diversity or plant composition more important to understand the structure of mollusk communities?, and (3) Do herbivorous mollusks have the potential to alter plant diversity, structure and composition in wildflower strips and, if so, what functional groups and species of plants are most affected?

## Methods

### Field manipulations

In spring (April–June) 2007, twelve wildflower strips were sown in field margins around the village of Grandcour, 10 km south of Lake Neuchatel in northwest Switzerland (479 m above sea level; coordinates: 46°52′N 06°56′E). Annual average temperature is 10.1°C, average annual precipitation amounts to 941 mm (Agrometeo 2011). The region is characterized by a mosaic of arable fields (intensive agriculture), grasslands and forests.

**Figure 1:**
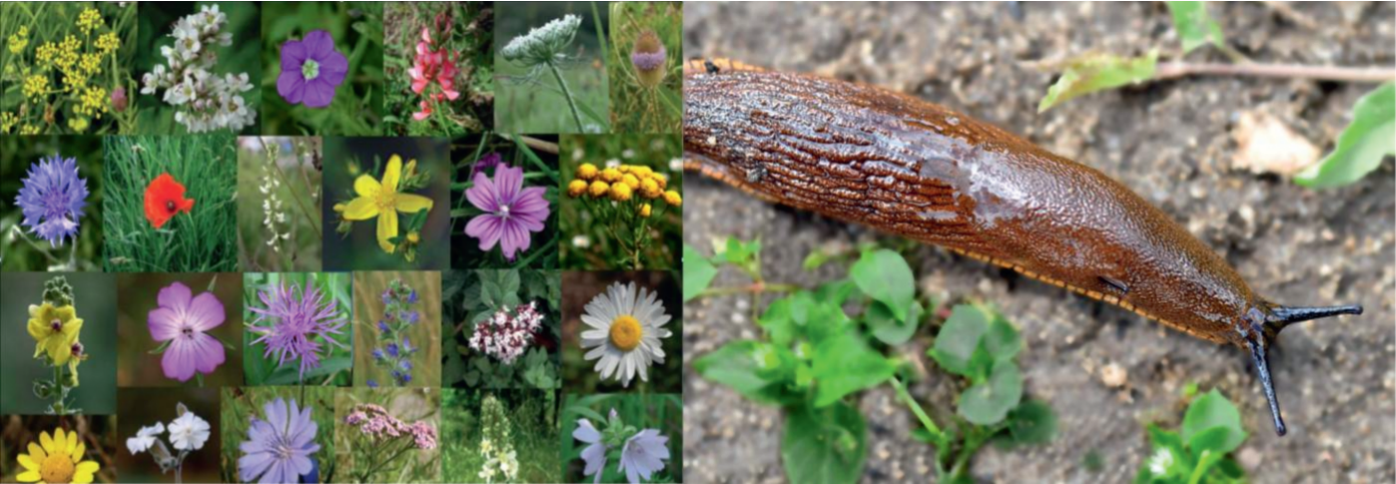
Photographs of the 24 plant species used in wildflower strips and of *Arion lusitanicus*, the most abundant mollusk species. Photo by H. Fabian.

**Figure 2:**
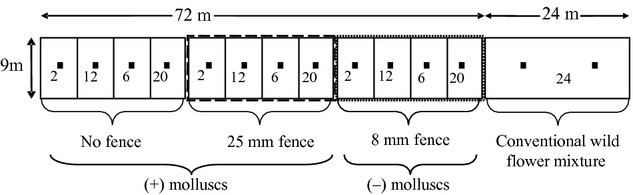
Layout of experimental wildflower strips with mollusk tile traps along the center (black squares). Numbers in the subplots indicate sown plant species number; dashed line represents a fence with 25 mm mesh size, dotted line with 8 mm mesh size.

Each wildflower strip was divided into four plots of 216 m^2^; one plot was sown with the full conventional wildflower mixture of 24 plants that farmers use in Central Switzerland (Günter 2000; see Fig. 1); the remaining three plots were randomly assigned to one of three treatments: (1) fence with 25-mm mesh size, (2) fence with 8-mm mesh size and molluscicide application and, (3) no fence. Within each of these three plots, we established four 6 × 9-m subplots differing in sown plant number (2, 6, 12, 20 sown species randomly assigned to the subplots, Fig. 2).

The plant species composition of each subplot was chosen by constrained random draw from a pool of 20 plant species with regard to equal frequency of occurrence in the 12 wildflower strips. We selected only species that belonged to the same functional group, tall herbs, to manipulate plant diversity only and not functional group diversity. We excluded two small herbs, *Legusia speculum-veneris* and *Fagopyrum esculentum*, the latter not native to central Europe, and two legumes, *Melilotus albus* and *Onobrychis viciifolia*, to prevent any possible bias through soil differences between subplots (Table S1; http://www.ufasamen.ch). Density of seeds corresponded to the official Swiss recommendations for sowing wildflower strips (Günter 2000). In contrast to other biodiversity experiments like Cedar Creek (Tilman et al. 1997), BIODEPTH (Otway et al. 2005) or The JENA Experiment (Roscher et al. 2004), our experimental plots were not weeded to avoid disturbance, with the exception of the problematic weeds *Cirsium arvense* and *Rumex obtusifolius*, and additionally in the first year (2007) *Chenopodium album* and *Amaranthus retroflexus* to prevent light competition during germination. Otherwise, plant communities were the result of self-assemblage following initial sowing.

The fencing treatment was intended to manipulate the densities of large vertebrate predators (reduced numbers in 25-mm and 8-mm fenced plots), and of micromammals and mollusks (reduced numbers in 8-mm fenced plots). However, only mollusks were successfully controlled in the 8-mm fenced plots (hereafter called “(–) mollusk”). Populations were reduced by application of 0.02 kg/m^2^ METAREX® (DE SANGOSSE, 47480 Pont du Casse, SA France), a 5% metaldehyde slug bait (Frank 1998b) every 2 weeks along the inside of the 8-mm fence, between mid-March and late October during the 3 years of the study. To avoid any confounding effect of metaldehyde pellets on seedling community composition and hence diversity, we applied molluscicides only along the inside of the 8-mm fence, and on the 30-cm wide central path (used to walk in the subplots), hence about 90% of the plot was unaffected. Furthermore, studies of the effect of metaldehyde on vegetation did not reveal any impact (Hector et al. 2004). Despite fencing and continuous live trapping of micromammals in 2007 and 2008, their density was not reduced. In 2009, a study of common voles (*Microtus arvalis*) and wood mice (*Apodemus sylvaticus*) revealed marginally greater abundances in 8-mm plots compared with 25-mm and control plots (replicated *G*-test, *P* = 0.108 and *P* = 0.098, respectively, Meyer 2011). Extensive observations during night and day revealed that vertebrate predators (foxes, hedgehogs and birds of prey) very rarely entered wildflower strips, even when unfenced (Gregoire Schaub, pers. comm. 2009). For this reason, we merged in this present study the treatments “25 mm fence” and “no fence,” hereafter called “(+) mollusk.” We found no effect of the fencing treatment on other animal species, notably on slug predators like carabid beetles (Y. Fabian unpubl. data).

### Plant data

In each subplot (Table S1), all plant species were identified and their individual percentage cover visually estimated using the standard method of Braun-Blanquet (Perner et al. 2005) in autumn 2007, 2008 and 2009. *Plant diversity* was characterized by species richness and the effective number of species. Species richness corresponds to the total number of plant species. The effective number of species (Jost 2006) is based on Shannon diversity and expresses species richness corrected for relative abundance – in our case, relative cover of plants.

*Vegetation structure* was characterized by the average vegetation height and plant biomass. Vegetation height was estimated as the average height of all plants of a subplot. Plant biomass was assessed with two different methods: in spring 15 April 2008–28 May 2008 we cut to ground level all plants in five squares of 30 cm per subplot. Samples were bagged and oven dried at 60°C to constant weight. We took the average weight of the five samples per subplot. In later periods this method became too work intensive because of the height of the vegetation (often >2 m). In early autumn 2008 and 2009, we measured the leaf area index (LAI) with a LAI-2000 (LI-COR Biosciences, Lincoln, Nebraska) at 24 random points in each subplot, and calibrated the method with five biomass samples from 8 and 16 subplots in 2008 and 2009, respectively. The resulting linear relationship (2008: Pearson product–moment correlation *r* = 0.89 and 2009: *r* = 0.87) was used to transform the average LAI values to plant biomass per subplot in g/m^2^.

The plant species were split into five functional groups: small herbs (<60 cm), tall herbs (>60 cm), legumes, grasses and small trees (Roscher et al. 2004). We used the number of species of the different plant functional groups and the cover of the plant functional groups in our analysis.

### Mollusk data

The abundance of mollusks was estimated using tiles (size 30 × 30 cm) as surface traps where mollusks could take shelter (Archard et al. 2004). We used 14 tiles per wildflower strip (Fig. 2), one in each subplot and two in the 24-species plot, laid on bare ground in August 2007. In the (–) mollusk plots, any mollusks and eggs found under the tiles were removed once in spring, summer, and autumn to maintain their exclusion. We sampled mollusks in September 2007, and in June and September 2008 and 2009. Species were determined following Kerney et al. (1983). Mollusks were counted and the length (*b* in cm) and width (*a* in cm) of each individual measured. Body volume (*V* in cm^3^) was calculated using the equation for a prolate spheroid:


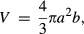


to analyze the average body volume per species, per plot, and subplot over the five different sessions. Additionally, we estimated the number of mollusk eggs under the tiles.

During the 3 years of the experiment, ants (Formicinae) increasingly established colonies underneath the tiles. The percentage cover of ant brood was estimated from digital photos as a measure of ant abundance.

### Climate data

To account for short-term effects of climate on mollusk abundance, the sampling was carried out on sunny days with air temperatures exceeding 12°C. We controlled for climate variability using climate measurements taken hourly from the Agrometeo website (Agrometeo 2011) for the meteorological station Delley, which lies 2–5 km from the experimental sites. For each sampling session, mean air temperature (*T* in °C), mean air humidity (*H* in %), and quadratic terms to model their optima (*T*^2^, *H*^2^) were included in all statistical models.

### Statistical analyses

All analyses were carried out using R version 2.12.0 (R Development Core Team 2012). Plant species richness was log transformed, percentage covers of plant species were square root transformed, and proportion of ant nest cover was arcsine square root transformed to correct for non-normality and heterogeneity of variance. Continuous explanatory variables were standardized to zero mean and unit variance using the function *scale* in vegan (Oksanen et al. 2011).

#### Effects of the vegetation on mollusk abundance

To test the effect of plant diversity and vegetation structure on mollusk abundance, we used the data from (+) mollusk plots only. The initial models included plant species richness, effective number of plant species, biomass, vegetation height, ant abundance, fence treatments, season and year as fixed effects and the 12 wildflower strips as random effects allowing for a random intercept. Weather conditions on the sampling day (*T*, *T*^2^, *H*, and *H*^2^) were always included in the models to control for short-term effects of the climate (for the weather effect on slugs, see Table S2). The total abundances of mollusks, mollusk eggs, and the individual slug species were modeled with the function *glmmadmb* in the package glmmADMB, fitting a zero-inflated negative binomial distribution (Zuur et al. 2009). We excluded the weather variables in the models for the slug eggs. All variables were included in the full model and the non-significant terms (*P* < 0.05) excluded in a backward stepwise procedure to select the simplest model. The function *glht* of the package multcomp (Hothorn et al. 2008) was used to compute the difference between years and thus allow multiple comparisons for parametric models. The analyses were performed firstly with plant species richness, secondly with species richness of the four plant functional groups (tall herbs, small herbs, grasses, and legumes), and thirdly with the cover of the functional groups. Note that we excluded the “tree” functional group in this analysis because there were only five species with very low cover (0.09%) in 2009 only. The body masses of the three most abundant mollusk species were analyzed with linear mixed effect models using *lme* (Pinheiro et al. 2011). Here we simplified full models by removing non-significant terms using the function *stepAIC* with forward and backward elimination (Venables and Ripley 2002).

#### Mollusk community structure

Canonical correspondence analysis (CCA) was performed in vegan (Legendre and Legendre 1998; Oksanen et al. 2011) to analyse the response of the mollusk community to plant diversity (species richness and effective number of species), vegetation structure (vegetation height and plant biomass) and plant composition (log transformed and scaled cover of the 30 most abundant plant species). Mollusk species that occurred in only one subplot (i.e., singletons) were excluded. Furthermore, we excluded all data from the (–) mollusk plots and included the 24-species plots. In all permutation tests between the environmental variables and mollusk community structure, 9999 constrained permutations were performed using the wildflower strips and sessions as block variables.

We compared the explanatory power of the three sets of vegetation descriptors (diversity, structure and composition) by partitioning the variation in the mollusk data (Hofer et al. 2000), using the function *varpart* in vegan (Oksanen et al. 2011). This application uses partial redundancy analysis (RDA) for community matrixes as independent variables and partial multiple regression analysis for vector-independent variables. Adjusted *R*^*2*^ values were calculated since it is the only unbiased method (Peres-Neto et al. 2006). We used the first two correspondence analysis axes of the plant composition as variables, resulting in equal numbers of explanatory variables for each environmental set (sets of variables with more descriptors would otherwise be comparatively overvalued in partial analyses). This allowed us to calculate the percentage of variance due exclusively and in common to the three groups of descriptors. To test significance of the exclusive fractions, we applied a test with 9999 permutations using the function *anova* in *varpart*.

#### Effects of mollusks on the vegetation

The effectiveness of the (–) mollusk treatment was tested by analysing the total mollusk abundance, abundance of the three most common species and the mollusk eggs, using linear mixed effect models (*lme*) in the package nlme (Pinheiro et al. 2011), with the three fence treatments and the sown plant number as fixed effects and the twelve wildflower strips as random effect. Again, the function *glht* (Hothorn et al. 2008) was used to compute the difference between treatments and years.

We then analysed the effects of mollusks on the vegetation. First, the effect of the mollusk treatment and of sown plant number on the plant species richness, effective number of species, vegetation height, plant biomass, and number of invading plant species (species other than those from the sown seed mixture) were analysed for the 3 years separately, with the twelve wildflower strips as random variables. The species richness and cover of the plant functional groups and the individual cover of plant species were then analysed for 2009, the year in which plant diversity differences between the mollusk treatments were significant. We analyzed only the 39 plant species that occurred in more than 20 of the 144 subplots and that had a mean cover >1% over all subplots in this year. We also analyzed the presence/absence data for these plant species using linear mixed effect models with a binomial function and logit link (*lmer* in the package lme4), again using the mollusk treatments and sown plant number as explanatory variables and the 12 wildflower strips as random variables. To correct for multiple testing, we computed *Q*-values on the basis of the 39 *P*-values correcting for the false discovery rate (FDR = No. of false positives/No. of significant tests) using the library q-value (Storey 2002). We fixed the tuning parameter *λ* to 0.0 (the most conservative value) for the presence/absence data and to a range between 0 and 0.9 for the cover data.

## Results

### Effects of the vegetation on mollusk abundance

A total of 2772 mollusks of seven different species were found under the 144 tiles in the 12 wildflower strips over the five sampling periods, with slugs of the species *A. lusitanicus* (Mabille)*, D. reticulatum* (Müller), and *D. panormitanum* (Lessona & Pollonera) accounting for 99% of all individuals (Table 1). The abundance of mollusks was significantly correlated with the number of mollusk species (*r* = 0.63, *P* < 0.001). There was large seasonal variation in slug abundance, with *A. lusitanicus* more common in spring and the two *Deroceras* species in autumn (Tables 1 and 2). Superimposed on this seasonal variation was a steady increase in the abundance of *A. lusitanicus*, whereas the abundances of the *Deroceras* species were highest in 2008 (Table 1).

**Table 1:** Absolute abundance and mean volume±SD (cm^3^) of mollusks and their eggs. Data come from 168 tiles for five trapping sessions

	2007	2008	2008	2009	2009	Total abundance	Mean volume±SD (cm^3^)
Species	Autumn	Spring	Autumn	Spring	Autumn		
*Arion lusitanicus*	10	164	100	457	337	1068	3.61±2.80
*A. rufus*	0	0	3	0	0	3	32.72±0.00
*Deroceras reticulatum*	364	57	473	9	241	1144	0.36±0.27
*D. panormitanum*	110	4	292	5	115	526	0.21±0.20
*Cepaea hortensis*	0	0	0	2	1	3	0.35±0.15
*C. nemoralis*	0	0	0	0	1	1	4.19±0.00
*Trichia hispida*	0	0	20	2	5	27	0.13±0.06
Mollusk eggs	100	0	6222	0	5493	11,815	–

**Table 2:** Results from the mixed effect models for the total mollusk abundance, abundance of the three most common slug species, and mollusk eggs

				Vegetation characteristics			Functional group richness				Functional group cover			
						
	Season	Year	Ant abundance	Veg. height	Plant biomass	Plant species richness	Tall herb species	Small herb species	Legume species	Grass species	Tall herb cover	Small herb cover	Legume cover	Grass cover
All mollusks	A>S	**09>**07>08	−0.75***	0.1*	–	−0.13†	–	−0.17*	–	–	–	−0.21**	–	–
*Arion lusitanicus*	S>A†	09>08**>07**	−1.00***	–	–	−0.47***	−0.30**	–	−0.36**	–	–	–	−2.30*	–
*Deroceras reticulatum*	A>S***	07>08>09	−0.30*	0.2*	–	0.19†	0.23**	–	–	–	–	–	–	−0.28**
*Deroceras panormitanum*	A>S***	**09>**07>08	−0.52*	–	0.48**	–	–	−0.23†	–	–	−0.22†	−0.49***	–	–
Mollusk eggs	A>S***	09**>**08>**07**	–	–	0.24**	–	–	–	–	–	–	–	–	–

Results from mixed effects models, as explained in the methods, with the slopes and significance for each response variable. Results for the variables that were excluded in a stepwise procedure from the full model are not shown (–). Plant diversity was log- and ant biomass arcsin sqrt transformed. Multiple comparisons for parametric models were performed - levels of factors shown in bold are significantly different from other levels (*P* < 0.05); Seasons: A, autumn; S, spring; Years: 2007, 2008, and 2009.

† *P* < 0.1,

* *P* < 0.05,

** *P* < 0.01, and

** ***P < 0.001.

**Figure 3:**
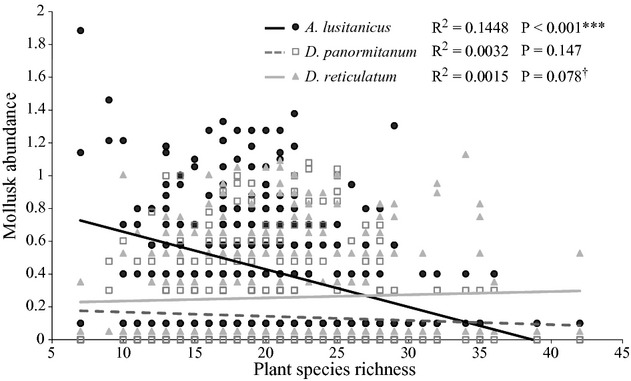
The relationship between plant species richness and mollusk abundance (log transformed) for *Arion lusitanicus* (black circles and line, to avoid over-plotting, a value of 0.1 was added), *Deroceras reticulatum* (light gray triangles and light gray line, a value of 0.05 was added) and *Deroceras panormitanum* (white squares and dashed line), over the two seasons of the years 2008 and 2009, within the (+) mollusk plots. Regression lines give the fitted linear model for each species. Significances calculated using linear mixed effect models.

Total mollusk abundance was negatively correlated with plant species richness and ant abundance and positively correlated with vegetation height (Table 2; Fig. 3). Mollusk abundance was also negatively correlated with the number of small herb species and their cover (Table 2). The three mollusk species showed different responses to the vegetation characteristics. The abundance of *A. lusitanicus* was negatively correlated with plant species richness in general and specifically with the number of legume and tall herb species (Table 2). The abundance of *D. panormitanum* was positively correlated with plant biomass, and negatively correlated with small herb cover, whereas *D. reticulatum* was positively correlated with vegetation height and the number of tall herb species, and negatively correlated with grass cover. The effective number of plant species was not correlated with abundance of any mollusk species. The abundance of mollusk eggs was positively correlated with plant biomass only.

The body mass of the three slug species was not influenced by plant species richness, effective number of species, vegetation height, or the treatments (Table S3). Only the body mass of *A. lusitanicus* was positively correlated with plant biomass.

### Mollusk community structure

**Figure 4:**
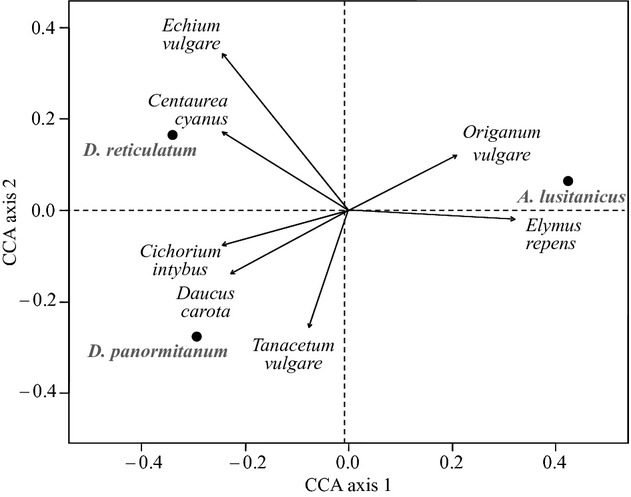
Canonical correspondence analysis biplot of mollusk community variation (boldface type in gray) dependent on plant species composition. Among the 30 most abundant plant species, only those significantly related to mollusk community composition are shown. Canonical correspondence analysis axis 1 eigenvalue = 0.195 and CCA axis 2 eigenvalue = 0.049.

**Figure 5:**
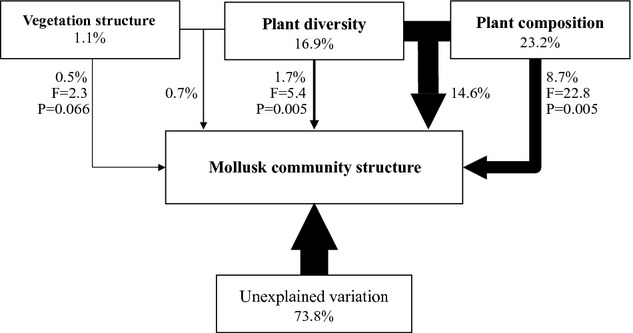
Variation partitioning of plant measures to explain mollusk community structure; percentages represent the explained variation. Two variables describe each vegetation characteristic: average vegetation height and plant biomass for vegetation structure, number of plant species and effective number of plant species for plant diversity, the first two ordination axes of the 30 most abundant plant species for plant composition. Note that vegetation structure and plant composition share no common variation, and that only the three independent fractions can be statistically tested.

Constrained ordinations revealed that the mollusk community was significantly influenced by *Centaurea cyanus*, *Cichorium intybus*, *Daucus carota*, *Echium vulgare*, *Tanacetum vulgare*, *Elymus repens* (all *P* < 0.005) and *Origanum vulgare* (*P* = 0.015; Fig. 4). The first five tall herb species were strongly positively associated with the two *Deroceras* species, whereas the last two species (a grass and a tall herb) were associated with the *Arion* species. The two slug genera separate along the first CCA axis, which explains the greatest part of the data (19.5%). The partial correspondence analysis of the determinants of the mollusk community showed that plant composition explained a total of 23.2% of the variation, and 8.7% exclusively (Fig. 5). It thus had much greater importance than plant diversity and plant structure, which explained a total of 16.9% and 1.1%, and exclusively 1.7% and 0.5%, respectively. The three sets of descriptors explained 26.2% of the total variation.

### Effects of mollusks on the vegetation

The reduction in mollusks by the fencing and molluscicide treatment was effective, with significantly lower abundances of mollusks and mollusk eggs in the (-) mollusk plots in 2008 and 2009 (Fig. S1).

**Figure 6:**
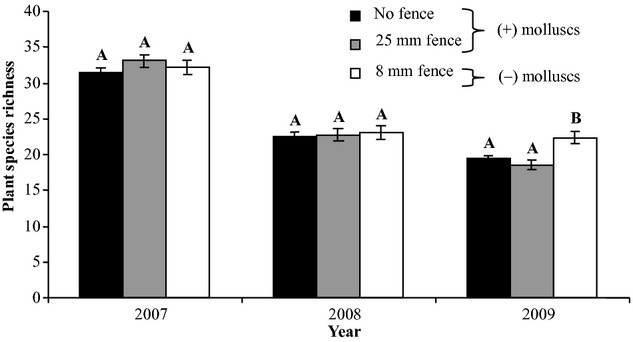
Average number of plant species for the three fence treatments for each year. Significant differences (*P* < 0.05) between fence treatments within a year are represented by different letters (A−B) calculated by multiple comparisons for parametric linear mixed effect models. Error bars represent the standard error of the mean from a total of 48 plots in the 12 wildflower strips.

**Figure 7:**
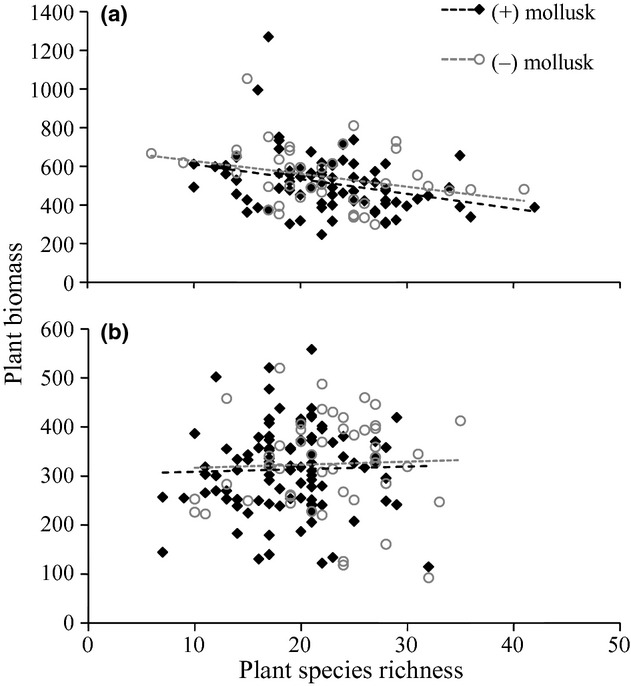
The relationship between plant species richness and biomass in (+) and (−) mollusk plots in the years (a) 2008 and (b) 2009.

**Figure 8:**
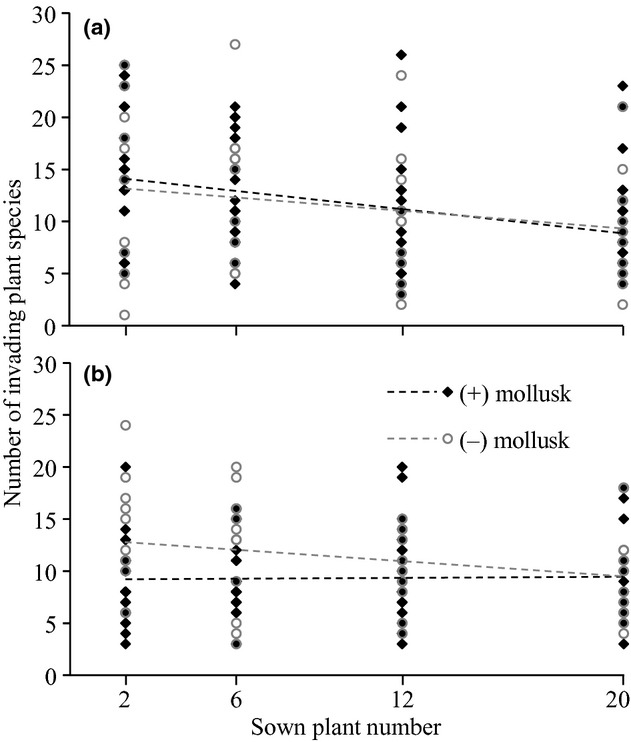
The relationship between the number of sown and invading plant species in (+) and (−) mollusk plots in the years (a) 2008 and (b) 2009.

Across treatments, the mean number of plant species per subplot decreased from 32.7 (±6.1; 19–45) in 2007 to 22.2 (±6.5; 6–42) in 2008 and 19.3 (±5.4, 7–35) in 2009 (standard deviation and range in brackets for the 12 strips, Fig. 6). The sown plant species number was positively correlated with the total plant species richness (*r* = 0.13, df = 280, *P* = 0.014), and with the effective number of species (*r* = 0.36, df = 280, *P* < 0.001). Plant species richness was significantly greater in the (–) mollusk compared with (+) mollusk plots in the year 2009 (*lme* value = 0.17; df = 130; *P* < 0.001). In 2007 and 2008, the treatments did not differ (Fig. 6). The relationship between plant species richness and biomass was not affected by mollusk herbivory in 2008 or 2009 (Fig. 7). The number of invading plant species (the subplots were not weeded) was negatively affected by the sown species number in 2008 and 2009 (*lme* value = −0.14 and −0.12, df = 128 and 129, respectively; *P* < 0.001 in both years) and was higher in the (–) mollusk treatment only in 2009 (*lme* value = 0.16, df = 129, *P* = 0.009), with no significant interaction between sown species number and treatment in both years (Fig. 8). There was no treatment effect on the effective number of species or vegetation height in any of the 3 years.

**Figure 9:**
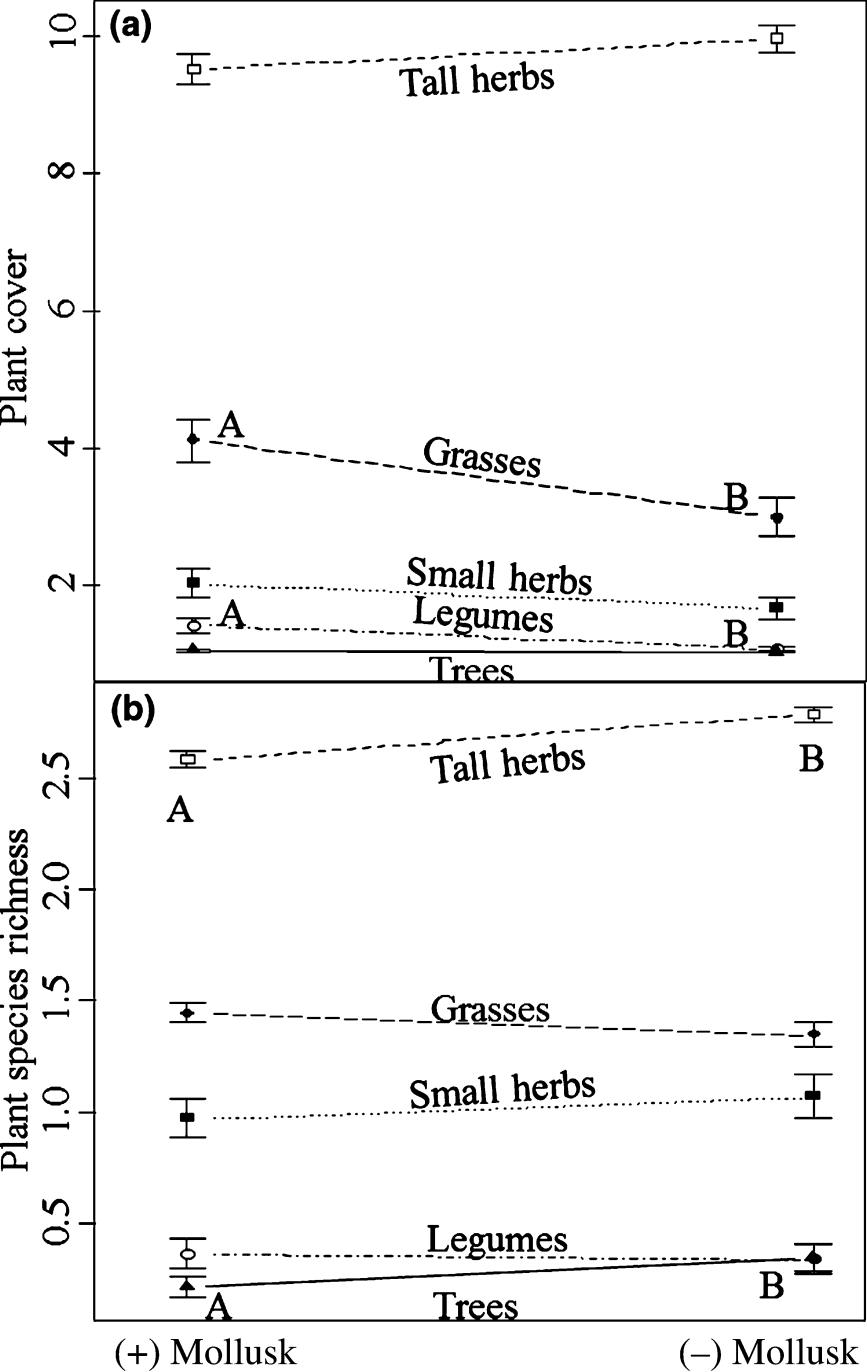
The effect of mollusks in the year 2009 on (a) plant cover (in %, square root transformed) and (b) plant species richness of five functional plant groups. Significant differences (*P* < 0.05) between treatments are represented by different letters. Symbols represent the means; error bars show the standard error.

In 2009, the year in which effects on plant species richness were seen, there were significantly more tall herb and tree species in the (−) mollusk than in the (+) mollusk plots, and the cover of grasses and legumes was lower in (−) mollusk plots (Fig. 9). When considering individual plant species, the presence and/or cover of eight tall herb species was significantly lower in (+) mollusk plots *(Achillea millefolium, Anthemis tinctoria*, *Cirsium arvense, Conyza canadensis, Echium vulgare, Daucus carota, Leucanthemum vulgare and Tanacetum vulgare)*, while the cover of *Dipsacus fullonum, Equisetum arvense, Dactylus glomerata, Lolium perenne* and *Trifolium repens* was significantly higher (Table 3).

**Table 3:** The effect of mollusks on the abundance or presence/absence of individual plant species

Plant species	(+) mollusk	(−) mollusk	Mollusk effect	*Q*-value
**Tall herbs**				
*Achillea millefolium*	0.64±0.49	0.88±0.33	–	0.014*
*Anthemis tinctoria*	0.55±0.50	0.77±0.42	–	0.033*
*Cirsium arvense^(c)^*	0.67±3.01	2.81±8.21	–	0.043*
*Conyza canadensis*	0.28±0.45	0.48±0.50	–	0.014*
*Echium vulgare*	0.23±0.42	0.52±0.50	–	0.014*
*Daucus carota*	0.43±0.50	0.65±0.48	–	0.014*
*Dipsacus fullonum^(c)^*	22.83±24.14	15.96±20.45	+	0.087†
*Leucanthemum vulgare*	0.51±0.50	0.73±0.45	–	0.014*
*Leucanthemum vulgare^(c)^*	4.30±9.21	12.23±21.12	–	0.003**
*Tanacetum vulgare*	0.61±0.48	0.83±0.38	–	0.037*
*Tanacetum vulgare^(c)^*	4.89±10.63	9.75±15.23	–	0.043*
**Grasses**				
*Dactylus glomerata^(c)^*	1.66±5.89	0.83±2.23	+	0.043*
*Equisetum arvense^(c)^*	0.71±2.73	0.03±0.10	+	0.077†
*Lolium perenne^(c)^*	3.30±9.72	0.90±2.80	+	0.046*
**Legumes**				
*Trifolium repens^(c)^*	0.48±1.61	0.08±0.52	+	0.098†

Arithmetic means ±SD based on data of the cover (indicated with “*(c)*” after the name) or the proportion of subplots in which plant species were present, with direction and significance of mollusk treatment in 2009. Mollusk effect: +, cover/abundance of the specific plant species increases with the presence of mollusks; −, cover/abundance of the specific plant species decreases with the presence of mollusks. *Q*-values are *P*-values from mixed effects models corrected for multiple tests (see methods section). We tested the 39 most abundant plant species; non-significant results were obtained for: *Centaurea jacea, Origanum vulgare, Elymus repens, Malva moschata, Epilobium sp., Hypericum perforatum, Arrhenaterum elatius, Apera spica-venti, Verbascum lychnitis, Silene latifolia, Holcus lanatus, Pastinaca sativa, Rumex obtusifolius, Taraxacum officinale, Rubus sp., Cichorium intybus, Malva sylvestris, Melilotus albus, Linaria vulgaris, Verbascum thapsus, Lactuca serriola, Setaria pumila, Phleum pratense, Plantago major, Plantago lanceolata* and *Sonchus asper*.

† *Q* < 0.10,

* *Q* < 0.05,

** *Q* < 0.01 and ***Q < 0.001.

## Discussion

In this semi-natural diversity experiment in wildflower strips over 3 years, we found evidence for a negative relationship between plant diversity and mollusk abundance, which leads to the conclusion that plant diversity can reduce the impact of herbivores (Root 1973). Moreover, plant identity had much greater importance than plant diversity as determinant of mollusk community composition. After 3 years, the highest plant diversity was found in the plots where mollusks were reduced, which is likely to result from selective feeding by mollusks leading to changes in plant composition and hence reduced plant diversity. Here we explore possible mechanisms behind our findings, outline their implications for biodiversity research in agro-ecosystems, and discuss experimental caveats of our study.

### Effects of the vegetation on mollusk abundance

We found a negative effect of plant species richness on slug abundance, which was mostly due to the lower abundance of *A. lusitanicus*. Hence we found evidence for the *resource concentration hypothesis* (Root 1973) for generalist herbivores. Our result is in contrast to Scherber et al. (2010a), who found that a higher plant species richness hosted more herbivores, consistent with the *more individuals hypothesis*. Their finding, however, concerned total herbivore species richness and abundance, and it would be interesting to analyze the relationships at a species or group level, because some taxa may behave differently. Indeed, we found that the abundance of *D. reticulatum* slightly increased with increasing plant – and especially tall herb – diversity (Dedov et al. 2006), which contrasts with the overall decrease driven by *A. lusitanicus*.

The plant functional groups had differential effects on the abundance of the slug species; however, the relationship between slug abundance and the cover or diversity of tall herbs, small herbs, legumes, and grasses was, with one exception, always negative. Other studies on generalist herbivores have similarly shown that plant functional identity was more important than plant diversity in determining the level of herbivory by grasshoppers (Pfisterer et al. 2003; Scherber et al. 2010b) and soil fauna (Birkhofer et al. 2011) in grasslands. These findings are supported by the variation partitioning analysis: we found that a large fraction of the variation remained unexplained, which can be expected for such eurytopic species; however, plant composition was significantly related to the distribution of slug communities, and accounted for by far the greatest exclusive fraction of the explained variation. Plant composition can thus have a substantial effect on so-called generalist herbivores (Scherber et al. 2010b).

Vegetation height and plant biomass were, in general, positively correlated with the abundance of mollusks, supporting the *plant architecture hypothesis* (Lawton 1983). There was also a positive effect of plant biomass, but not of species richness, on the abundance of slug eggs. It suggests that vegetation structure and especially biomass is important for slugs when choosing egg-laying sites. The importance of vegetation structure on mollusk abundance has been shown for wetlands (Horsak et al. 2011), but rarely in agroecosystems within intensive agriculture (but see Dedov et al. 2006).

### Effects of mollusks on the vegetation

Our treatment reducing the abundance of mollusks (predominantly the large species *A. lusitanicus*) revealed a substantial impact of slugs on the species richness and composition of wildflower strips after only 2 years. Total productivity, measured by biomass, was however not affected. Plant species richness was substantially greater when mollusks were reduced, which is in line with the finding that the establishment of invading plants was hindered in (+) mollusk plots. Specifically, there were more species of tall herbs and tree seedlings in (–) mollusk plots, at the expense of grass and legume cover, which is in accordance with other diversity studies (Allan and Crawley 2011) and feeding experiments using *A. lusitanicus* (Briner and Frank 1998) and *D. reticulatum* (Keller et al. 1999; Hensgen et al. 2011). For instance, *A. lusitanicus* shows clear preferences for annual plant species that are sown in wildflower strips and in crop fields over naturally occurring legumes and grasses (Briner and Frank 1998). Thus, these unpalatable plants occupy empty niches created by selective mollusk herbivory on certain tall herb species, a phenomenon that has been documented not only for mollusks in grassland (Allan and Crawley 2011), but also for grasshoppers (Scherber et al. 2010b). In the (+) mollusk plots, we found a higher abundance of *Dipsacus fullonum*, *Equisetum arvense*, *Trifolium repens, Lolium perenne* and *Dactylus glomerata*. Our findings for these species are supported by feeding (Hanley et al. 1995) and grassland experiments (Allan and Crawley 2011), providing evidence that they occupy empty niches produced by slug grazing on specific tall herbs.

A very encouraging result for farmers is the strong negative effect of mollusk grazing on *Cirsium arvense*, an agricultural pest plant that requires expensive and time-consuming herbicide control (Marshall et al. 2003; Ziska et al. 2004) and that can become abundant in wildflower strips. *Cirsium* is known to be affected by mollusk grazing in feeding experiments (Briner and Frank 1998), and we indeed found that *Cirsium* can be significantly reduced by slug herbivory, despite a high availability of other palatable herbs.

Long-term field experiments in perennial grassland have yielded highly variable results, with examples in which there is no effect of aboveground herbivores on plant diversity or biomass (Stein et al. 2010), where there is negative effect of mollusks on plant diversity but a positive effect on plant biomass (Allan and Crawley 2011), or *vice versa* (Buschmann et al. 2005). This highlights the importance of performing long-term diversity experiments in the specific natural environments under concern, to draw conclusions about herbivore effects and conservation aspects.

### Experimental caveats

The desired effect of the 8-mm fencing treatment was to decrease the abundance of major herbivores in the system, namely slugs and small rodents (common voles and wood mice). As in other field experiments, the exclusion of any trophic group is likely to be incomplete (Stein et al. 2010) even when pesticides, as in our study, are applied frequently at high dosage, or exclusion constructions are carefully built. As mentioned in the Methods, in the same 12 study strips, Meyer (2011) found more rodents in (−) mollusk compared with (+) mollusk plots (average±SD: 2.1±1.5 and 1.5±1.2 captured rodents per plot, respectively). We can assume that this small numerical difference in rodent abundance made at most a minor contribution to the observed differences in vegetation. In contrast, there were much greater differences in mollusk abundances between (–) and (+) mollusk plots (average±SD: 4.4±2.1 and 18.4±4.3 captured individuals, respectively). Moreover, the difference was most marked for the largest species, *A. lusitanicus*. Thus we suggest that the major vegetation differences between mollusk treatments derived from mollusk grazing. Pellet analyses showed that the common vole and the wood mouse have contrasting food preferences to mollusks; they prefer grasses and legumes (Lantova and Lanta 2009; Meyer 2011). Thus, the observed decrease in cover of both functional groups in the (–) mollusk plots may be partly attributed to an increased grazing pressure from rodents, reinforced by stronger competition with tall herbs released from herbivory by slugs.

In order to avoid disturbance to the plants, we did not search the vegetation exhaustively for mollusks or carry out soil sampling, which are considered the most reliable methods for mollusk sampling (South 1964). Surface trapping using tiles has limitations and does not estimate absolute abundances. Moreover, it shows a bias for slugs with higher body mass (Archard et al. 2004; Cordoba et al. 2011). However, the method is fully adequate to estimate differences in slug abundances between subplots and it enabled us to monitor the mollusk community development over a period of 3 years, without drastically reducing abundances as would have been the case had pitfall traps been used.

During the 3 years of the experiment, ants increasingly established colonies underneath the tiles. At the end of the experiment in autumn 2009, 57 of 168 tiles (35%) were colonized by *Lasius niger* (Linnaeus) and 2 tiles by *Lasius flavus* (Fabricius). We found a positive effect of plant species richness and especially of legume, tall herb and small herbs on ant abundance (*lme* value = 0.05; *P* = 0.005, Y. Fabian, unpubl. data). This effect can be expected, as a higher diversity of plants provides a higher diversity of resources in the form of aphids and seeds (Boulton et al. 2005; Scherber et al. 2010a; Haddad et al. 2011). Ant abundance was strongly negatively correlated with slug abundance, and in particular for *A. lusitanicus*. For this reason, ant abundance was accounted for in our analyses; we also reanalysed the data excluding all tiles with ants, which did not yield different results. Thus, we can assume that ants do not mediate the effect of plant diversity on slug abundance. Surprisingly, (–) mollusk plots had significantly fewer ants than (+) mollusk plots. The application of molluscicide and/or vegetation effects could explain this negative impact on ants.

## Conclusion

Our diversity experiment showed that plant diversity, structure, and composition can have substantial effects on mollusk abundance and composition. In particular, the agricultural pest species *A. lusitanicus* was less abundant in more diverse habitats. Species-rich communities thus appear to be more resistant against generalist herbivores, as has been suggested for specialist herbivores by the *resource concentration hypothesis* (Root 1973). Plant species composition was the most important determinant of the overall composition of the gastropod community. This finding was supported by the differential effect of the five plant functional groups. Thus, selective feeding and active habitat choice in mollusks might be the reason for lower abundances in diverse habitat patches. It should therefore be possible to optimize the species composition of wildflower strips to reduce their attractiveness to slugs, while maintaining their role in the promotion of ecosystem services such as pollination and the preservation of biodiversity in farmland.

We also provide evidence for a significant decrease in plant species richness caused by mollusks, resulting in a compositional change in the vegetation. Future studies on ecosystem functioning should therefore avoid focusing only on singular descriptors of vegetation, such as simple diversity or biomass, but in addition measure vegetation composition components and species traits. Also, the negative impact of slugs was evident only after 2 years; to show the combined effect of herbivory and plant species richness on biomass, future studies might have to run for longer time.

Our results highlight the importance of differentiating the effects of plant diversity and composition on different herbivore species in ecosystem functioning research. They also demonstrate the protective role of plant biodiversity against generalist herbivores, which can in turn negatively impact plant diversity on a longer term, driving the system along a “low plant diversity – high mollusk abundance” trajectory.

## Supporting Information

**Figure S1.** Abundances of *A. lusitanicus, Deroceras panormitanum, D. reticulatum* and mollusk eggs in the mollusk treatments.

**Table S1.** Wildflower species composition and cover.

**Table S2.** Mollusk abundance in relation to the meteorological data.

**Table S3.** Body mass analysis of the three most abundant mollusk species.
