# Supplementary material for: Diversity protects plant communities against generalist molluscan herbivores
Source: Ecol Evol. 2012 Aug 31;2(10):2460–73. doi: 10.1002/ece3.359 (PMC3492773; doi:10.1002/ece3.359)
Supplement: Supplementary file 1 [file ece30002-2460-SD1.pdf]

Diversity protects plant communities against generalist molluscan herbivores

Yvonne Fabian, Nadine Sandau, Odile T. Bruggisser, Patrik Kehrli, Alexandre Aebi, Rudolf P. Rohr, Russell E. Naisbit and Louis-Félix Bersier

Appendix

Table S1. Wildflower species composition and cover.

Table S2. Mollusc abundance in relation to the meteorological data.

Table S3. Body mass analysis of the three most abundant mollusc species.

Figure S1. Abundances of the molluscs *A. lusitanicus*, *D. panormitanum, D. reticulatum* and mollusc egg abundance in the mollusc treatments.

Table S1) Wildflower species composition and cover. Internal = sown plants from the Swiss wildflower mixture. External = other species that were found in the experimental wildflower strips. Functional groups: TalH = tall herbs; SmlH = small herbs; Leg = legumes, SmlT = small trees and Grass = grasses. The numbers of plots the plant occurred in and the mean cover (%) are given for the years 2007, 2008 and 2009. Internal plant species in grey were not used in the fence experiments. All 24 internal plant species were sown in the conventional wildflower mixture plots.

Plant species	Internal or External	Functional group	Number of plots in			Mean cover in		
			2007	2008	2009	2007	2008	2009
Achillea millefolium L.	Int	TalH	96	120	113	3.4	6.8	4.4
Agrostemma githago L.	Int	TalH	76	24	16	3.2	0.2	0.02
Anthemis tinctoria L.	Int	TalH	83	100	98	4.0	6.4	6.4
Centaurea cyanus L.	Int	TalH	91	10	0	8.0	0.1	0
Centaurea jacea L.	Int	TalH	99	118	116	6.6	9.8	14.4
Cichorium intybus L.	Int	TalH	101	113	59	18.7	17.6	1.0
Daucus carota L.	Int	TalH	107	121	79	9.6	6.8	1.1
Dipsacus fullonum L.	Int	TalH	96	117	140	8.6	11.1	19.3
Echium vulgare L.	Int	TalH	88	105	53	7.6	6.3	0.3
Fagopyrum esculentum Moench	Int	SmlH	19	3	0	0.6	0	0
Hypericum perforatum L.	Int	TalH	47	94	96	0.2	1.4	3.0
Legusia speculum-veneris (L.) Chaix	Int	SmlH	10	0	0	0.04	0	0
Leucanthemum vulgare Lam.	Int	TalH	86	95	90	2.6	5.8	7.1
Malva moschata L.	Int	TalH	87	99	111	3.3	3.7	4.9
Malva sylvestris L.	Int	TalH	103	97	64	3.8	1.7	0.8
Melilotus albus Medik.	Int	Leg	20	24	20	0.1	0.05	0.8
Onobrychis viciifolia Scop.	Int	Leg	11	10	7	0.09	0.06	0.02
Origanum vulgare L.	Int	TalH	68	98	108	0.6	2.4	7.6
Papaver rhoeas L.	Int	TalH	90	40	16	0.9	0.4	0.2
Pastinaca sativa L.	Int	TalH	40	51	75	0.2	0.8	1.5
Silene latifolia Poir.	Int	TalH	90	117	108	1.8	3.4	2.2
Tanacetum vulgare L.	Int	TalH	96	109	106	2.2	4.0	6.1
Verbascum lychnitis L.	Int	TalH	45	65	90	0.5	1.2	1.9
Verbascum thapsus L.	Int	TalH	77	91	61	2.0	1.6	0.5
Acer pseudoplatanus L.	Ext	SmlT	1	5	6	0	0	0.001
Aethusa cynapium L.	Ext	TalH	21	23	0	0.07	0.2	0
Agrostis stolonifera L.	Ext	Grass	0	47	0	0	0.8	0
Althea officinalis L.	Ext	TalH	1	0	3	0	0	0
Amaranthus lividus L.	Ext	TalH	20	0	0	0.03	0	0
Amaranthus retroflexus L.	Ext	TalH	99	10	0	0.6	0.007	0
Anagallis arvensis L.	Ext	SmlH	71	35	10	0.3	0.2	0.003
Apera spica-venti (L.) P.Beauv.	Ext	Grass	9	1	68	0.007	0.05	1.9
Arrhenatherum elatius (L.) P.Beauv.	Ext	Grass	2	8	43	0.009	0.1	2.6
Borago officinalis L.	Ext	TalH	0	0	4	0	0	0
Brassica sp. 1	Ext	TalH	1	0	0	0.001	0	0
Brassica sp. 2	Ext	TalH	9	0	0	0.08	0	0
Brassica napus L.	Ext	TalH	33	1	0	0.4	0	0

Table S1 *continued*<br>Plant species	Internal or External	Functional group	Number of plots in 2007	2008	2009	Mean cover in 2007	2008	2009
Bromus sp.	Ext	Grass	4	0	0	0.03	0	0
Campanula patula L.	Ext	TalH	0	1	0	0	0.02	0
Capsella bursa-pastoris (L.) Medik.	Ext	SmlH	107	12	0	1.0	0.03	0
Cardamine pratensis L.	Ext	TalH	0	0	5	0.0	0	0.006
Centaurium erythraea Rafn	Ext	SmlH	0	0	1	0	0	0
Cerastium sp.	Ext	SmlH	22	22	5	0.1	0.1	0.0
Chamomilla suaveolens	Ext	TalH	69	0	0	1.6	0	0
Chaenorhinum minus (L.) Lange	Ext	SmlH	18	8	0	0.02	0.02	0
Chamomilla recutita (L.) Rauschert	Ext	TalH	41	17	3	0.2	0.06	0.001
Chenopodium album L.	Ext	TalH	106	23	8	1.1	0.7	0.05
Chenopodium polyspermum L.	Ext	TalH	106	18	5	3.5	0.4	0.0
Chenopodium sp.	Ext	TalH	26	23	13	0.08	2.5	0.1
Circea lutetiana L.	Ext	SmlH	22	33	41	0.3	0.5	1.5
Cirsium arvense (L.) Scop.	Ext	TalH	0	0	1	0	0	0.001
Cirsium vulgare (Savi) Ten.	Ext	TalH	0	0	3	0	0	0
Clematis vitalba L.	Ext	SmlH	0	0	1	0	0	0
Convolvulus arvensis L.	Ext	SmlH	5	13	16	0.003	0.1	0.1
Conyza canadensis (L.) Cronquist	Ext	TalH	0	29	54	0	0.3	1.2
Cornus sanguinea L.	Ext	TalH	3	0	15	0	0	0.05
Coronilla sp.	Ext	Leg	0	0	1	0	0	0.006
Crepis biennis L.	Ext	TalH	1	3	1	0	0	0
Dactylis glomerata L.	Ext	Grass	11	27	75	0.04	0.3	1.9
Deschampsia flexuosa (L.) Trin.	Ext	Grass	0	0	3	0	0	0.1
Digitaria sanguinalis (L.) Scop.	Ext	Grass	2	3	0	0.0	0	0
Echinochloa crus-galli (L.) P.Beauv.	Ext	Grass	119	71	27	6.0	1.7	0.03
Elymus repens (L.) Gould	Ext	Grass	7	51	95	0.05	1.5	5.9
Epilobium hirsutum L.	Ext	TalH	0	0	3	0	0	0
Epilobium sp. 1	Ext	TalH	21	100	93	0.01	0.8	3.4
Epilobium sp. 2	Ext	TalH	0	0	91	0	0	3.5
Equisetum arvense L.	Ext	Grass	29	24	24	1.2	0.9	0.5
Erigeron annuus (L.) Pers.	Ext	TalH	0	1	8	0	0	0
Euphorbia amygdaloides L.	Ext	TalH	1	0	0	0	0	0
Euphorbia exigua L.	Ext	SmlH	3	0	0	0.002	0	0
Euphorbia helioscopia L.	Ext	TalH	6	10	4	0.01	0.02	0
Euphorbia stricta L.	Ext	TalH	0	13	1	0	0.2	0
Fallopia convolvulus (L.) A. Löwe	Ext	SmlH	2	33	8	0.002	0.1	0.007
Festuca sp.	Ext	Grass	0	2	10	0	0.001	0.2
Filaginella uliginosa (L.) Opiz	Ext	SmlH	19	6	0	0.05	0.008	0
Fragaria sp.	Ext	SmlH	0	1	0	0	0	0
Galinsoga ciliata (Raf.) S.F.Blake	Ext	SmlH	16	3	0	0.09	0	0
Galium album Miller	Ext	TalH	0	5	9	0	0.001	0.03
Galium aparine L.	Ext	SmlH	25	14	15	0.1	0.04	0.02
Geranium dissectum L.	Ext	SmlH	4	0	0	0.001	0	0
Geranium rotundifolium L.	Ext	SmlH	3	11	9	0.009	0.05	0.01
Geum urbanum L.	Ext	TalH	0	0	5	0	0	0

Table S1 *continued*<br>Plant species	Internal or External	Func-tional group	Number of plots in 2007	2008	2009	Mean cover in 2007	2008	2009
Glechoma hederacea L.	Ext	SmlH	3	6	7	0.001	0.001	0.1
Grass sp.1	Ext	Grass	0	4	0	0	0.2	0
Grass sp.2	Ext	Grass	9	0	0	0.1	0	0
Grass sp.3	Ext	Grass	5	0	0	0.0	0	0
Grass sp.4	Ext	Grass	2	0	0	0.002	0	0
Gypsophila muralis L.	Ext	SmlH	2	0	0	0	0	0.0
Helianthus annuus L.	Ext	TalH	11	0	0	0.1	0	0
Holcus lanatus L.	Ext	Grass	3	17	58	0.01	0.2	1.6
Hypochoeris radicata L.	Ext	TalH	0	0	2	0	0	0.02
Juglans regia L.	Ext	SmlT	5	20	25	0.001	0.01	0.08
Juncus bufonius L.	Ext	Grass	19	4	5	0.1	0.03	0.0
Juncus sp.	Ext	Grass	2	4	4	0.002	0.03	0.02
Kickxia elatine (L.) Dumort.	Ext	SmlH	25	2	0	1	0.006	0
Kickxia spuria (L.) Dumort.	Ext	SmlH	18	13	7	0.04	0.2	0.04
Lactuca serriola L.	Ext	TalH	0	11	7	0	0.4	0.4
Lamiaceae	Ext	SmlH	1	0	0	0	0	0
Lamium amplexicaule L.	Ext	SmlH	0	2	2	0	0.001	0
Lamium purpureum L.	Ext	SmlH	18	17	6	0.009	0.005	0
Linaria vulgaris L.	Ext	SmlH	11	17	22	0.02	0.1	0.6
Lolium perenne L.	Ext	Grass	18	71	67	0.3	0.9	2.5
Lotus corniculatus L.	Ext	Leg	23	10	16	0.2	0.001	0.01
Lythrum salicaria L.	Ext	TalH	0	0	5	0	0.0	0
Malva neglecta Wallroth	Ext	TalH	2	0	0	0.008	0	0
Medicago lupulina L.	Ext	Leg	3	5	2	0.0	0.001	0.001
Medicago sativa L.	Ext	Leg	0	1	2	0.0	0	0
Mentha arvensis L.	Ext	SmlH	0	3	3	0	0.06	0.05
Mercurialis annua L.	Ext	TalH	26	12	5	0.3	0.02	0.04
Myosotis arvensis (L.) Hill	Ext	SmlH	13	10	8	0.008	0.003	0.007
Oenothera biennis L.	Ext	TalH	0	1	1	0	0	0
Orobanche sp.	Ext	SmlH	0	3	2	0	0	0.001
Oxalis stricta L.	Ext	SmlH	20	34	12	0.06	0.1	0.2
Phacelia tanacetifolia Benth.	Ext	TalH	2	0	0	0.008	0	0
Phleum pratense agg.	Ext	Grass	6	12	17	0.01	0.05	0.3
Phragmites australis (Cav.) Trin.	Ext	Grass	0	0	1	0	0	0
Plantago lanceolata L.	Ext	SmlH	15	25	22	0.08	0.2	0.3
Plantago major L.	Ext	SmlH	101	111	37	1.5	1.4	0.3
Poa annua L.	Ext	Grass	91	27	3	1.8	0.5	0.05
Polygonum aviculare L.	Ext	SmlH	27	42	9	0.2	1.1	0.02
Polygonum mite Schrank	Ext	TalH	33	26	6	0.4	0.1	0.001
Polygonum sp.	Ext	TalH	36	5	0	0.6	0.001	0
Populus alba L.	Ext	SmlT	0	0	3	0	0	0
Portulaca oleracea L.	Ext	SmlT	2	0	0	0.002	0	0
Potentilla reptans L.	Ext	SmlH	2	4	6	0	0.006	0.007
Prunella vulgaris L.	Ext	SmlH	0	4	1	0	0	0
Ranunculus repens L.	Ext	SmlH	5	22	17	0.07	0.3	0.08

Table S1 *continued*<br>Plant species	Internal or External	Functional group	Number of plots in 2007	2008	2009	Mean cover in 2007	2008	2009
Rubus sp.	Ext	SmlH	10	7	11	0.07	0	1.0
Rumex obtusifolius L.	Ext	TalH	68	65	77	1.1	0.7	1.3
Rumex sp.	Ext	TalH	4	0	0	0.0	0	0
Sagina apetala Ard.	Ext	SmlH	1	1	0	0.0	0	0
Salix alba L.	Ext	SmlT	2	8	26	0	0.007	0.007
Salix caprea L.	Ext	SmlT	0	1	4	0	0	0.0
Scrophularia nodosa L.	Ext	TalH	2	1	6	0	0	0.001
Senecio vulgaris L.	Ext	TalH	25	19	0	0.06	0.001	0
Setaria pumila (Poir.) Schult.	Ext	Grass	36	46	25	0.8	0.7	0.4
Sinapis alba L.	Ext	TalH	0	1	0	0	0	0
Solanum nigrum L.	Ext	SmlH	46	9	1	0.4	0	0
Solidago canadensis L.	Ext	TalH	0	0	2	0	0	0
Sonchus arvensis L.	Ext	TalH	80	69	0	0.5	0.9	0
Sonchus asper (L.) Hill	Ext	TalH	0	15	35	0	0.09	0.2
Sonchus oleraceus L.	Ext	TalH	12	10	2	0.03	0.03	0
Stellaria media (L.) Vill.	Ext	SmlH	104	60	17	2.3	0.7	0.02
Taraxacum officinale Wigg	Ext	SmlH	66	67	51	2.4	1.1	1.2
Taraxacum sp.	Ext	SmlH	1	0	1	0.03	0	0
Thlaspi arvense L.	Ext	SmlH	4	0	0	0.009	0	0
Trifolium arvense L.	Ext	Leg	1	0	0	0	0	0
Trifolium campestre Schreb.	Ext	Leg	2	0	0	0.009	0	0
Trifolium pratense L.	Ext	Leg	19	28	11	0.1	0.5	0.03
Trifolium repens L.	Ext	Leg	72	60	36	1.2	0.8	0.3
Trifolium sp.1	Ext	Leg	2	0	0	0.0	0	0
Trifolium sp.2	Ext	Leg	1	0	1	0.0	0	0.006
Tripleurospermum inodorum L.	Ext	TalH	4	0	4	0.02	0	0.03
Triticum sp.	Ext	Grass	9	18	12	0.03	0.03	0
Urtica dioica L.	Ext	TalH	1	5	5	0	0	0
Veronica verna L.	Ext	SmlH	5	0	0	0.003	0	0
Verbena officinalis L.	Ext	TalH	0	5	14	0	0.006	0.03
Veronica persica Poir.	Ext	SmlH	94	58	37	2.3	0.2	0.05
Veronica serpyllifolia L.	Ext	SmlH	4	4	1	0	0.007	0
Vicia hirsuta (L.) Gray	Ext	Leg	6	5	5	0.009	0	0.01
Vicia sativa L.	Ext	Leg	7	0	2	0.01	0	0
Viola arvensis Murray	Ext	SmlH	17	18	6	0.1	0.02	0.001

Table S2) Mollusc abundance in relation to the meteorological data. Parameters of Generalized linear mixed effect models with a correlation structure between sessions and the traps within the wildflower strips. Significance levels at: **.** P < 0.1, * P < 0.05, ** P < 0.01 and *** P < 0.001

	Air temperature (°C)	Optimum air temperature (°C)^2	Air humidity (%)	Optimum air humidity (%)^2
Molluscs	-0.329	0.000	1.274 **	-1.466 ***
Arion lusitanicus	-0.199	0.355	0.471 **.**	-0.414 **.**
Deroceras reticulatum	-0.277	-0.091	0.857 *	-1.008 **
Deroceras panomitanum	-0.034	0.026	0.024	-0.025

Table S3) Body mass analysis of the three most abundant mollusc species

Linear mixed-effects model fit by maximum likelihood for slug body mass

Fixed effects:
treatment + plant species richness + effective number of species + biomass + veg. height + year + season

Data: *Arion lusitanicus*

Random effects: Formula: ~1 |wildflower strip

	Intercept	Residuals
s.d.:	3.71	9.75

Fixed effects:	Value	Std.Error	DF	t-value	p-value	
Intercept	26.83	4.97	211	5.40	0.000	
25 mm fence	1.06	1.45	211	0.73	0.467	
8 mm fence	-1.86	2.01	211	-0.93	0.356	
Plant species richness	1.10	0.75	211	1.47	0.143	
Effective number of species	-0.67	0.92	211	-0.73	0.468	
Plant Biomass	3.44	1.03	211	3.33	0.001	**
Vegetation height	-0.19	0.87	211	-0.22	0.826	
Year 2008	-3.01	5.06	211	-0.59	0.553	
Year 2009	-8.12	4.83	211	-1.68	0.094	.
Season spring	-11.43	1.81	211	-6.33	<0.001	***

Standardized Within-Group Residuals:

Min	Q1	Med	Q3	Max
-2.60	-0.49	-0.12	0.35	5.57

Number of Observations: 232
Number of Groups: 12

Table S3 *continued*

Data: *Deroceras reticulatum*

Random effects: Formula: ~1 |wildflower strip

	Intercept	Residuals
s.d.:	0.19	0.84

Fixed effects:	Value	Std.Error	DF	t-value	p-value	
(Intercept)	1.61	0.18	245	9.12	0.000	
25 mm fence	-0.12	0.13	245	-0.93	0.352	
8 mm fence	0.00	0.14	245	0.01	0.992	
Plant species richness	0.03	0.07	245	0.42	0.678	
Effective number of species	0.01	0.07	245	0.16	0.876	
Plant biomass	0.03	0.09	245	0.32	0.746	
Vegetation height	-0.08	0.08	245	-1.02	0.308	
Year 2008	-0.77	0.22	245	-3.53	0.001	**
Year 2009	-0.40	0.20	245	-1.99	0.048	*
Season spring	0.00	0.18	245	-0.03	0.980	

Standardized Within-Group Residuals:

Min	Q1	Med	Q3	Max
-1.62	-0.57	-0.11	0.33	6.70

Number of Observations: 266
Number of Groups: 12

Data: *Deroceras panormitanum*

Random effects: Formula: ~1 |wildflower strip

	Intercept	Residuals
s.d.:	0.12	0.45

Fixed effects:	Value	Std.Error	DF	t-value	p-value	
(Intercept)	0.68	0.14	137	4.70	0.000	
25 mm fence	0.03	0.09	137	0.39	0.701	
8 mm fence	-0.14	0.10	137	-1.32	0.188	
Plant species richness	-0.03	0.05	137	-0.63	0.533	
Effective number of species	0.03	0.04	137	0.66	0.509	
Plant biomass	-0.04	0.06	137	-0.66	0.510	
Vegetation height	0.03	0.06	137	0.56	0.579	
Year 2008	-0.28	0.17	137	-1.69	0.094	.
Year 2009	0.23	0.15	137	1.52	0.131	
Season spring	-0.31	0.19	137	-1.62	0.107	

Standardized Within-Group Residuals:

Min	Q1	Med	Q3	Max
-1.86	-0.64	-0.09	0.371	4.80

Number of Observations: 158
Number of Groups: 12

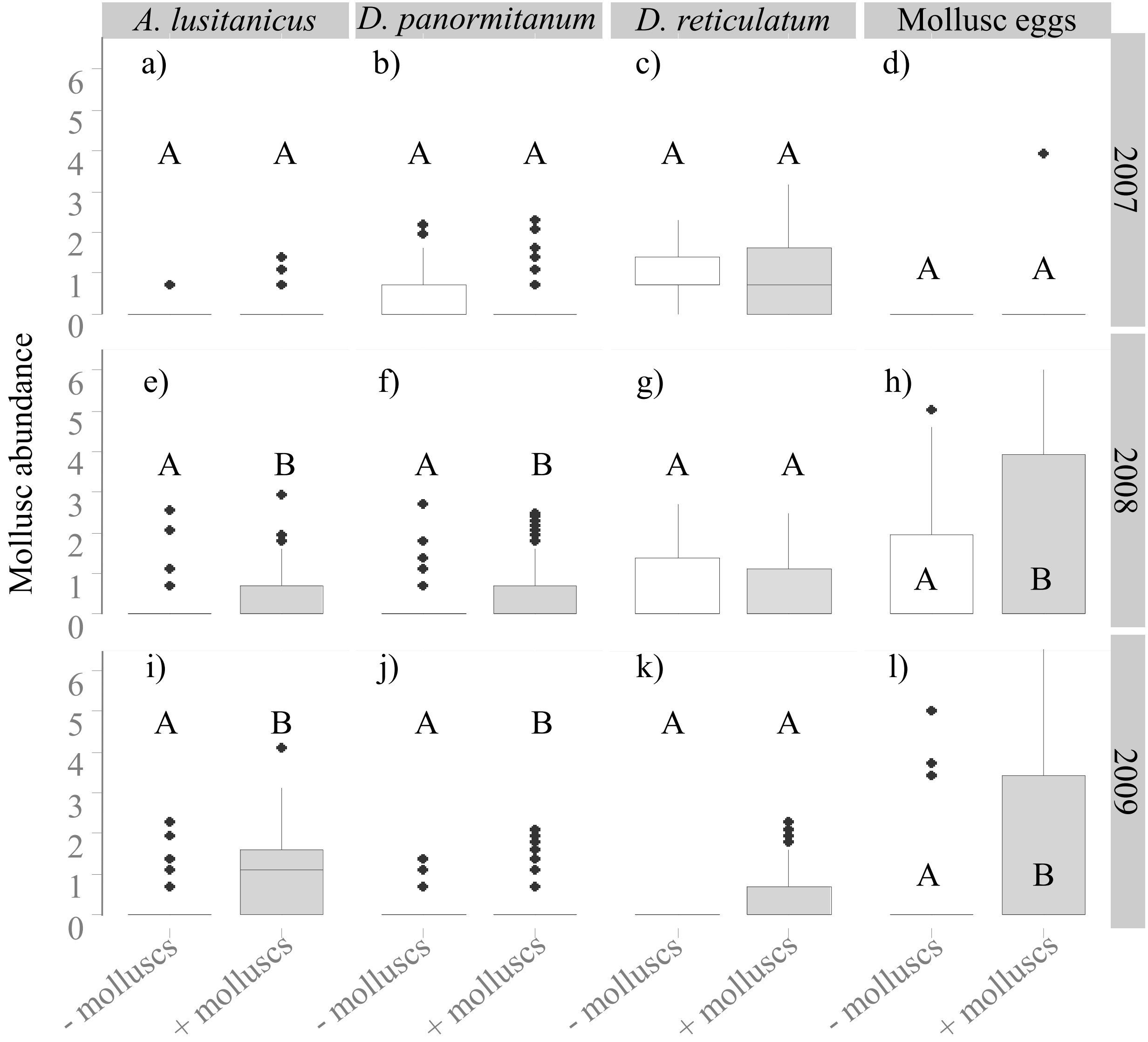

Figure S1) Abundances of the molluscs *A. lusitanicus*, *D. panormitanum, D. reticulatum* and mollusc egg abundance (left to right) in 2007, 2008 and 2009 (top to bottom) in the mollusc treatments (- white and + grey). The twelve graphs a) - l) show logarithmic means (of two seasons and twelve wildflower strips) of slug abundances for each of the three species and the mollusc eggs. Different letters A-B show significantly different slug abundances ($P < 0.05$) within one species, between the mollusc treatments in the corresponding year analysed by linear mixed effect models. The box plots represent the median, the upper and lower borders of the boxes 25 and 75 quartiles and lines give the maximum and minimum. Dots indicate abundances that are considered as outliers.
