# Supplementary figures and images for: Diversity protects plant communities against generalist molluscan herbivores
Source: Ecol Evol. 2012 Aug 31;2(10):2460–73. doi: 10.1002/ece3.359 (PMC3492773; doi:10.1002/ece3.359)

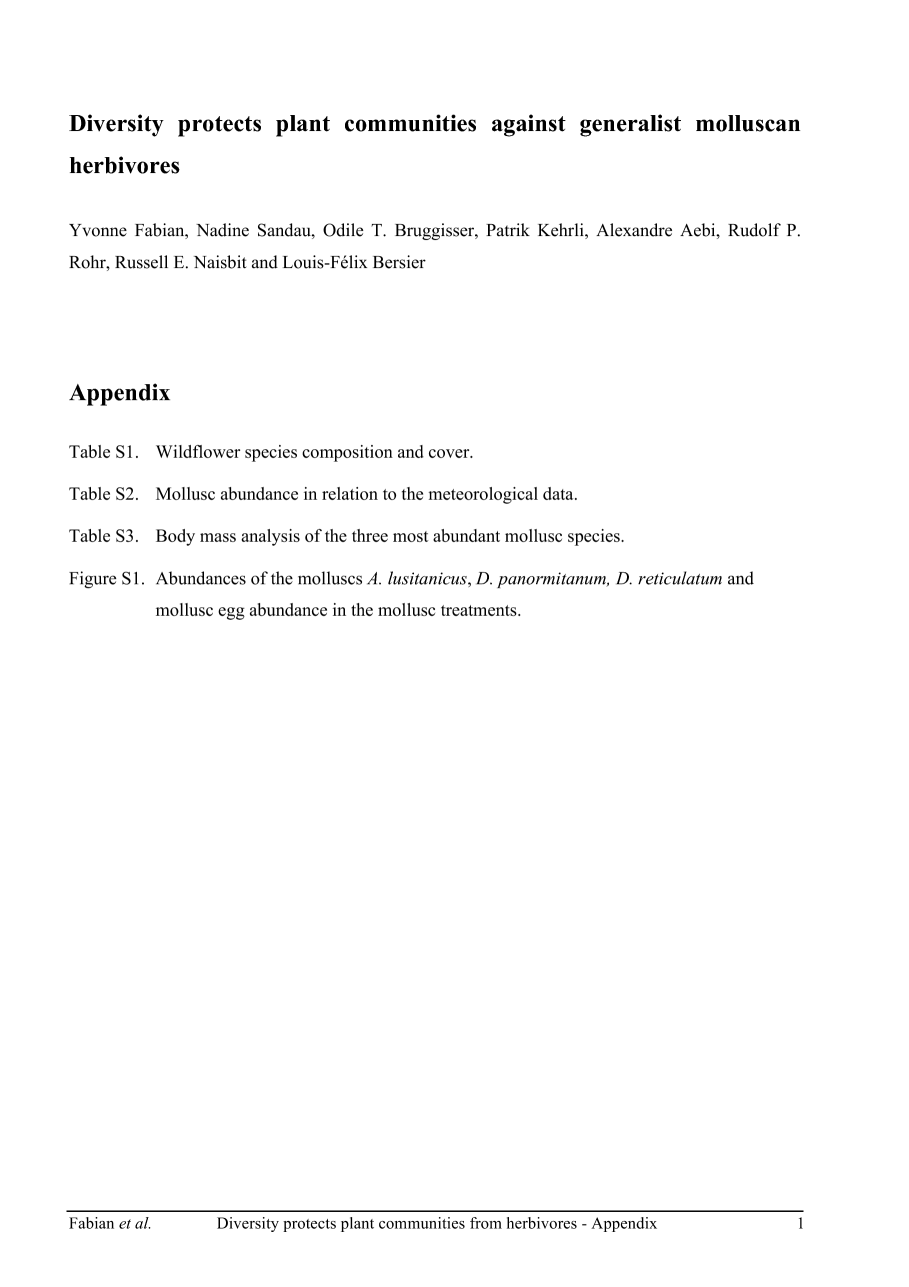

Supplement: Supplementary file 2 [file ece30002-2460-SD2.png]
